# Molecular and neural adaptations to neuromuscular electrical stimulation; Implications for ageing muscle

**DOI:** 10.1016/j.mad.2020.111402

**Published:** 2021-01

**Authors:** Yuxiao Guo, Bethan E Phillips, Philip J Atherton, Mathew Piasecki

**Affiliations:** MRC-Versus Arthritis Centre for Musculoskeletal Ageing Research and National Institute of Health Research, Biomedical Research Centre, School of Medicine, University of Nottingham, UK

**Keywords:** Neuromuscular electrical stimulation, Ageing, Muscle, Atrophy, Denervation, Motor unit remodelling

## Abstract

One of the most notable effects of ageing is an accelerated decline of skeletal muscle mass and function, resulting in various undesirable outcomes such as falls, frailty, and all-cause mortality. The loss of muscle mass directly leads to functional deficits and can be explained by the combined effects of individual fibre atrophy and fibre loss. The gradual degradation of fibre atrophy is attributed to impaired muscle protein homeostasis, while muscle fibre loss is a result of denervation and motor unit (MU) remodelling. Neuromuscular electrical stimulation (NMES), a substitute for voluntary contractions, has been applied to reduce muscle mass and functional declines. However, the measurement of the effectiveness of NMES in terms of its mechanism of action on the peripheral motor nervous system and neuromuscular junction, and multiple molecular adaptations at the single fibre level is not well described. NMES mediates neuroplasticity and upregulates a number of neurotropic factors, manifested by increased axonal sprouting and newly formed neuromuscular junctions. Repeated involuntary contractions increase the activity levels of oxidative enzymes, increase fibre capillarisation and can influence fibre type conversion. Additionally, following NMES muscle protein synthesis is increased as well as functional capacity. This review will detail the neural, molecular, metabolic and functional adaptations to NMES in human and animal studies.

## Highlights

• Muscle atrophy and functional declines observed with advancing age can be minimized via various NMES protocols.
• Animal models have shown that NMES induces motor axon regeneration and promotes axonal outgrowth and fibre reinnervation.
• The activation of BDNF-trkB contributes to promotion of nerve growth and survival and mediates neuroplasticity.
• NMES is able to regulate muscle protein homeostasis and elevate oxidative enzyme activity.

## 1 Introduction

An accelerated loss of skeletal muscle mass and function is a predictable accompaniment of ageing (Convertino, 1990; Cruz-Jentoft and Sayer, 2019; Vandenborne et al., 1998). This physiological process of muscle atrophy, weaker strength and a slower gait speed is collectively defined as sarcopenia (Rosenberg, 1997). Its functional associations and consequences include a high incidence of falls and fractures (Chalhoub et al., 2015; Hida et al., 2016; Landi et al., 2012; Yeung et al., 2019), mobility disorders (Marcus et al., 2012), frailty (Cesari et al., 2014; Wilson et al., 2017), all-cause mortality (Cruz-Jentoft et al., 2019), and substantial individual and social financial burden (Beaudart et al., 2014; Bruyère et al., 2019).

The contraction of skeletal muscles depends on the regulation and coordination of its neural input, which also undergoes significant adaptations in response to ageing. The final motor nervous system associated with muscle contraction is the motor unit (MU), which consists of an efferent motor neuron and all of the muscle fibres it innervates (Liddell and Sherrington, 1925). A decreased number of MUs has been identified in a number of aged muscles in humans, leaving some muscle fibres denervated (Piasecki et al., 2016) and resulting in the loss of muscle mass and functional decrement (Luff, 1998). The denervation of muscle fibres not only occurs in the process of ageing, but also occurs in some neuropathological disorders, such as amyotrophic lateral sclerosis (Gonzalez Calzada et al., 2016) and autosomal recessive spinal muscular atrophy in children (Kolb and Kissel, 2015).

Resistance exercise has proven to be an effective countermeasure to neuromuscular decrements, including in the elderly (Binder et al., 2005; Frontera et al., 1988; Leenders et al., 2013; Suetta et al., 2008; Tsuzuku et al., 2018; Verdijk et al., 2009). However, certain situations such as injury and/or prolonged bed rest render resistance exercise intervention an unachievable option, particularly in elderly populations, and neuromuscular electrical stimulation (NMES) has been applied as a surrogate to mitigate or treat muscle mass and strength decreases (Kern et al., 2014). The contraction of skeletal muscle triggered by electrical stimulator devices is a result of the depolarisation of motor neuron axons and their branches. This can be achieved in several ways, including stimuli on the superficial muscle belly via self-adhesive surface electrodes, and directly over a motor nerve (Enoka et al., 2019; Mortimer and Bhadra, 2004a). Depending on the goals of the intervention, NMES, composed of stimulation-rest cycles, is provided within a range of frequencies to generate muscle tetany and muscle contraction, over periods of weeks or months (Doucet et al., 2012).

**Fig. 1:**
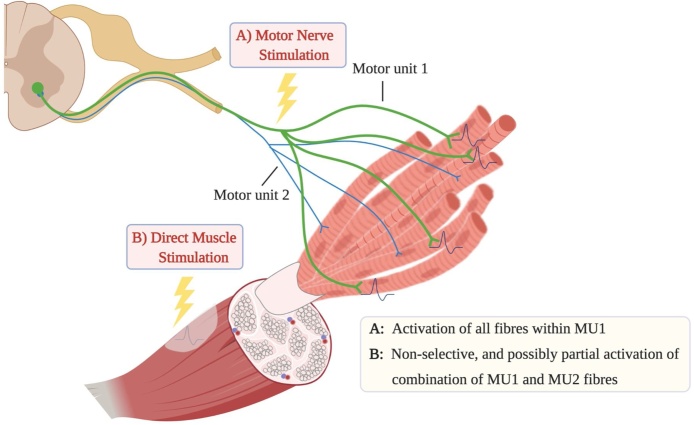
Motor unit recruitment following neuromuscular electrical stimulation. A) Motor nerve stimulation activates superficial motor neurons, and all fibres within that motor unit; B) Direct muscle stimulation non-selectively activates the muscle fibres in close proximity to the stimulating electrodes, which may not include complete motor units.

According to the Henneman size principle, the recruitment of MUs in voluntary contractions presents a temporally asynchronous, spatially diffused pattern from slow twitch muscle fibres to fast twitch fibres, from small MUs to larger ones (Henneman et al., 1965), whereas the recruitment pattern via NMES is temporally synchronized, spatially fixed, and non-selective (Gregory and Bickel, 2005; Semmler, 2002), evidenced by early recruitment of a large number of fatigable fast twitch muscle fibres. Unlike motor nerve stimulation activating all muscle fibres within a MU, direct muscle stimulation non-selectively activates fibres in close proximity to the stimulating electrodes, which may not include complete MUs (Fig. 1). The excitation of muscle or nerve is largely dependent on proximity to stimulating electrodes, with axon depolarisation also dependent on membrane resistance (Kiernan and Bostock, 2000; Mortimer and Bhadra, 2004b). Put simply, NMES induces a poorly defined and non-physiological order of MU recruitment.

Although NMES has been applied as a clinical treatment to maintain or enhance muscle strength and relieve muscle tension, the assessment of the effectiveness is mostly based on a small number of parameters, has been applied largely in younger cohorts, and fails to account for multiple adaptations i.e. molecular responses at the muscle fibre and those that are deemed clinically relevant such as motor control and balance. Importantly, its mechanisms on the peripheral motor nervous system and adaptive changes it produces at the neuromuscular junction (NMJ) remain largely unexplored in humans.

As such, the purpose of this review is to explore the physiology of NMES in terms of both neural and muscular adaptations in animal and human studies, with the intention of providing a more informed foundation for future studies of ageing.

## 2 Neural plasticity and NMES

Electromyography (EMG), the recording of electrical activity from muscle (Mills, 2005), is widely accepted and used to study MU structure and function (Heckman and Enoka, 2012; Piasecki et al., 2018a; Piasecki et al., 2020). These methods range in complexity but largely involve surface based and/or intramuscular measures, able to generate representations of the ionic exchange across the muscle fibre membrane. The summated action potential of a single MU is referred to as a MU potential (MUP) and represents the summation of depolarization from fibres innervated by the same axon, within the recording range of the electrode. Features of the MUP, such as amplitude, duration, phases, etc., reflect aspects of the MU, such as size, fibre density and complexity, which are routinely used in clinical applications (Dhand, 2014; Katirji, 2007). The compound muscle action potential (CMAP), elicited via maximal electrical stimulation, has also been used as an estimate of muscle excitability from involuntary contractions (Araújo et al., 2015) and is associated with physical frailty in elderly populations (Swiecicka et al., 2020, 2019). Calculating motor unit number estimates (MUNE) has largely been applied to track the motor unit loss in different pathologies (Gooch et al., 2014) or compare across populations i.e. young and old (Piasecki et al., 2016, 2018b; Power et al., 2013).

Involuntary muscle contractions generated by NMES have been considered as a potential strategy to limit the denervation-induced muscle fibre loss and maintain muscle function (Kern et al., 2017), with involvement of efferent and afferent pathways (Gondin et al., 2006, 2005). The NMES-elicited depolarisation of the motor axons transmits descending signals to the motor endplate directly (Maffiuletti et al., 2018) and in parallel the sensory neurons depolarise and transmit ascending signals obtained from direct depolarisation, muscle spindles, Golgi tendon organs and cutaneous receptors (Burke et al., 1983) to the spinal cord (Bergquist et al., 2011; Collins, 2007). The repetitive activation of the afferents generates a somatosensory input, resulting in both central and peripheral involvement (Blickenstorfer et al., 2009; Han et al., 2003; Smith et al., 2003).

The exploration of NMES-induced plasticity of the peripheral nervous system lacks convincing evidence from human experimental data, and most results are derived from animal models (Al-Majed et al., 2000b; Brushart et al., 2002; Johnson and Connor, 2011). For example, electrical stimulation was applied to the thigh muscles of rats following sciatic nerve sectioning, and to a large extent induced reinnervation of the dennervated muscle fibre (Eken and Gundersen, 1988; Vivó et al., 2008). Similarly, a greater MUP size was observed in rabbits following high-intensity NMES, suggesting that NMES improved the regenerative capacity of motor neurons (Nix and Hopf, 1983).

**Fig. 2:**
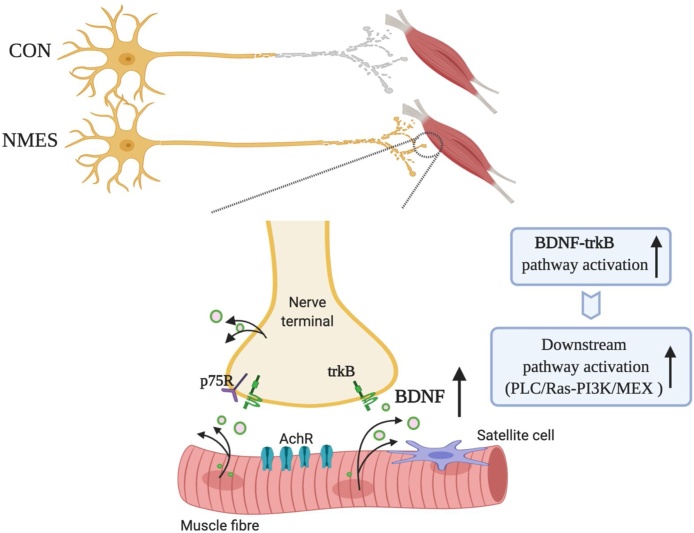
Nerve regeneration and muscle reinnervation. Neuromuscular electrical stimulation (NMES) accelerates axonal outgrowth and the reinnervation process at the neuromuscular junction (NMJ), mediated by brain-derived neurotrophic factor (BDNF) through the tropomyosin-related kinase receptor B (trkB), and its downstream pathways.

Successful nerve regeneration and muscle reinnervation following NMES ultimately depends on the homeostatic plasticity of the NMJ (Fukazawa et al., 2013). MU expansion requires axonal sprouting, including terminal and nodal sprouting (Hoffman, 1950), and the ability of the original intact axons to form additional neuromuscular connections at synapses (Brown et al., 1981; Slater, 2017; Tomori et al., 2010). A continual supply of neurotrophic factors, such as brain-derived neurotrophic factor (BDNF), glial-derived neurotrophic factor (GDNF) and nerve growth factor (NGF) (Gordon, 2010; Höke et al., 2006), contributes to the axonal regeneration process following NMES. Of all neurotrophic factors, BDNF is the most predominant molecule involved in axonal regeneration (Zhang et al., 2000) and its expression has been proven to be elevated following NMES (Al-Majed et al., 2000a; Wenjin et al., 2011; Willand et al., 2016). Increased BDNF expression can promote axonal sprouting via the trkB signalling pathway (Greising et al., 2015; Mantilla et al., 2014) (Fig. 2), followed by the initiation of downstream signalling pathways (Hurtado et al., 2017; Pradhan et al., 2019), including BDNF-PLC/Ras-PI3K/MEX pathways, and once the axonal sprout reaches the muscle fibre and completes the formation of the intact NMJ, the role of BDNF adapts, now binding to the p75 receptors on the nerve terminal to inhibit continued axonal growth and re-establish the functional connections at the NMJ (Gordon et al., 2003).

A certain intensity and duration of voluntary exercise contributes to the enhancement of BDNF levels, and the upregulation of BDNF has a positive correlation with blood lactate content (Boyne et al., 2019; Dinoff et al., 2017; Ferris et al., 2007; Müller et al., 2020; Schiffer et al., 2011), known to increase during exercise. NMES has been reported to elevate the levels of BDNF and lactate in both animal and human studies (Dalise et al., 2017; Hamada et al., 2004), with circulating BDNF levels reaching a similar level or even a higher level as in voluntary exercise (Kimura et al., 2019; Miyamoto et al., 2018), and the increase of lactate was positively correlated with the increase of BDNF (Kimura et al., 2019). The upregulation of BDNF levels and the lactate concentration following low-intensity muscle involuntary contractions is likely due to the non-selective motor unit recruitment pattern, with NMES activating additional fast-twitch MUs (Watanabe et al., 2014). Even if the electrical stimulation may not have the reversed recruitment order, its non-selective principle has the potential to activate a larger proportion of high-threshold motor units.

Given the mounting evidence highlighting adaptations to the peripheral motor system as a major contributor to loss of muscle mass and function in humans (Filippo et al., 2017; Gonzalez-Freire et al., 2014; Hepple and Rice, 2016; Piasecki et al., 2018b), data derived from animal experiments has provided a mechanistic foundation for exploring the benefits of NMES as a pre/rehabilitation strategy to promote neural adaptations in human studies.

## 3 Morphological effects of NMES

Muscle atrophy and the inability to produce efficient force are the main consequences of neuromuscular deficiencies. Muscle architecture is evaluated from physiological or anatomical cross-sectional area (CSA), muscle fibre length and pennation angle (Lieber and Fridén, 2000). Although data are equivocal because of the different populations studied, various characteristics of NMES protocols and the inclusion of resistance exercise and/or nutrition, collectively it is probable that NMES positively influences total muscle size in humans (Karlsen et al., 2020; Sillen et al., 2013). Eight weeks high-frequency NMES (75 Hz) applied to both vastus lateralis and vastus medialis muscles in healthy elderly resulted in a significant increase of CSA (Filippo et al., 2017). NMES over 4 months also induced an increase in knee extensors CSA, and a greater increase was observed when combined with voluntary exercise (Benavent-Caballer et al., 2014). At the level of individual muscle fibres, histochemical and morphological results following nine weeks NMES revealed that an increase of diameter and percentage was observed in fast-type muscle fibres, while the diameter of slow fibres was decreased (Kern et al., 2014; Zampieri et al., 2015).

The distribution and classification of muscle fibres in humans is based on the content of three predominantly identified myosin heavy chain (MHC) isoforms, consisting of type 1, 2A and 2X (Schiaffino and Reggiani, 2011). An upregulation of MHC-2A and a downregulation of MHC-I expression have been reported in the elderly received eight weeks NMES (Mancinelli et al., 2019). The variability of the plasticity of MHC following NMES may be attributed to variations in stimulation levels (Minetto et al., 2013) and/or variability in the non-selective order of MU recruitment (Gregory and Bickel, 2005). Notwithstanding neural input and the range of applied protocols, there are also several factors that contribute to the conversion of MHC phenotype, including an individual’s habitual physical activity levels (sedentary or active) which could influence baseline MHC isoforms distribution (Gondin et al., 2011).

Combined with the adaptive changes in muscle architecture, the adaptations of muscle fibre capillaries following NMES is of interest due to their role in substrate delivery, for instance, oxygen, which is directly related to the muscle fibre size (Bosutti et al., 2015). Evidence showed that the capillary proliferation and muscle fibre growth followed a similar time course in the skeletal muscles in humans (Verdijk et al., 2016), suggesting a positive relationship between capillarisation and muscle fibre hypertrophy. Although the data exploring the adaptations of capillary supply is scant in healthy elderly, it’s found that high-frequency NMES improved the capillarisation of the muscles and preceded the conversion of muscle fibre phenotype (Perez et al., 2002), highlighting the importance of angiogenesis and muscle fibre capillarisation, particularly in older muscle (Prior et al., 2016).

## 4 Molecular effects of NMES

### 4.1 Muscle protein synthesis

An imbalance between muscle protein synthesis (MPS) and muscle protein breakdown (MPB) leads directly to individual fibre atrophy, and total muscle atrophy (Wilkinson et al., 2018). Protein nutrition and exercise are regarded as the main countermeasures to attenuate or prevent muscle atrophy (Brooks et al., 2008; Jordan et al., 2014; Wall et al., 2015). Regardless of nutritional intake, even some low volume physical exercise is able to maintain skeletal muscle mass (Ferrando et al., 1997; Oates et al., 2010), further highlighting the potential of NMES as an interventional therapy.

In healthy older individuals, five days of bed rest with NMES and protein supplementation resulted in no significant decrease in muscle mass (Reidy et al., 2017), which would be expected to occur without intervention (Snijders et al., 2019). Similarly Dirks et al., identified the effectiveness of NMES combined with pre-sleep protein intake on MPS in older adults. Prior to 20 g protein feeding, a 70-min single bout of NMES was conducted unilaterally on the lower limb, and muscle biopsies after 4 h showed no difference in myofibrillar MPS between the stimulated and control leg (Dirks et al., 2016). However, the same NMES protocol including 40 g rather than 20 g protein intake, showed an increase in muscle protein synthesis 8 h post feeding, suggesting that metabolic responses to NMES are sensitive to nutritional intervention and are time dependent (Dirks et al., 2017). Further to this, a single session of NMES was performed in elderly individuals with type 2 diabetes, who are known to be more susceptible to muscle loss and functional decline (Park et al., 2006), and was shown to elicit a large increase (27 %) in MPS (Wall et al., 2012). Four weeks of home-based daily NMES in patients with knee osteoarthritis resulted in increased muscle fibre size, which was matched with a heightened rate of MPS (Gibson et al., 1989).

According to the limited available data on NMES and MPS, NMES used directly as an isolated strategy or as an adjuvant to nutritional (protein-based) interventions can increase MPS and as such, help mitigate the anabolic resistance commonly observed in ageing muscle (Breen and Phillips, 2011).

### 4.2 Metabolic adaptations

The term exercise commonly refers to two modalities; aerobic endurance training and anaerobic resistance training, or a combination of these two. Each of these modalities is associated with distinct physiological adaptations including intramuscular metabolic changes (Bell et al., 2000), such as alterations of active oxidative enzymes with endurance training (Carter et al., 2001) and active glycolytic enzymes with resistance training (Tesch et al., 1989).

The most common enzymatic reaction in human body is the tricarboxylic acid cycle (Krebs cycle), in which citrate synthase (CS) is paramount. Findings from four studies which applied low-frequency NMES for 4–10 weeks demonstrated an increase in the activity levels of CS by 9 %–31 % (Gauthier et al., 1992; Nuhr et al., 2003; Thériault et al., 1996; Theriault et al., 1994), with greater increases in women when compared to men (Gauthier et al., 1992). Moreover, Theriault and colleagues examined the response of metabolic enzymes to different lengths of NMES intervention and showed that the activity level of CS increased after four weeks, with no further change after an additional four weeks (Theriault et al., 1994). Similarly, isocitrate dehydrogenase (IDH), another enzyme involved in Krebs cycle, was also increased following eight-weeks of high-frequency NMES (Gondin et al., 2011). The premise of achieving energy production from the Krebs cycle is the beta oxidation of fatty acids (Rasmussen and Wolfe, 1999), and the levels of 3-Hydroxylacyl-CoA dehydrogenase (HADH) increased by up to 30 % following low-frequency NMES (Gauthier et al., 1992; Theriault et al., 1994). However, this outcome was reversed when applying high-frequency NMES, showing a decreasing trend (Gondin et al., 2011). Findings from the same study did however report an increase in the second step of beta oxidation; a greater level of Enoyl CoA hydratase (Gondin et al., 2011). With regards to other oxidative enzymes, succinate dehydrogenase, cytochrome c oxidase and pyruvate dehydrogenase all increased following several weeks NMES stimulation (Gauthier et al., 1992; Perez et al., 2002; Theriault et al., 1994).

Unlike the significantly increased levels of oxidative enzymes shown in most experiments, glycolytic enzymes reportedly remained unchanged or decreased after NMES. For example, although there was a decrease in the activity level of glyceraldehyde 3-phosphate dehydrogenase (GAPDH) following ten weeks NMES intervention (Nuhr et al., 2003), a separate study showed that it did not change after six weeks low-frequency NMES (Gauthier et al., 1992). However, another glycolytic enzyme, phosphofructokinase (PFK) showed a mild decrease in activity with the six-week intervention, specifically decreasing in men by 10 %, with little change in women (Gauthier et al., 1992). High-frequency NMES in active individuals and sedentary young individuals revealed differing outcomes for beta-enolase, a glycolytic enzyme involved in the process of glycolysis of 2-phosphoglycerate to phosphoenolpyruvate (PEP), with an increase in the active young individuals, but no change in sedentary group (Gondin et al., 2011).

However, the majority of studies looking at metabolic enzyme activity have focused on low-frequency NMES, with limited available data following high-frequency NMES. Additionally, although some studies applying chronic low-frequency NMES have shown some benefits on oxidative capacity, the severity and duration of some protocols may render them impractical for elderly populations.

## 5 Functional effects of NMES

NMES has proven to play an important role in minimising functional declines caused by ageing (Amiridis et al., 2005; Bax et al., 2005). Muscle torque in the elderly increased over several weeks NMES intervention as determined by maximal isometric voluntary contractions (MVC) (Caggiano et al., 1994; Kern et al., 2014; Mignardot et al., 2015; Paillard et al., 2004). In addition to measuring muscle force directly, a number of functional tests are commonly recommended and applied in clinical practice or research, such as the timed up and go test (TUG) (Podsiadlo and Richardson, 1991), the Berg balance scale (BBS) (Berg et al., 1992) and the short physical performance battery (SPPB) (Guralnik et al., 1994), among others. NMES-based interventions have proven to shorten the time for completing activities of daily living, for example climbing stairs, potentially indicating a higher level of muscle strength and power of the lower extremities in older adults (Kern et al., 2014; Langeard et al., 2020; Zampieri et al., 2015). Using a similar stimulation frequency and doubling the number of training sessions, NMES improved balance gait speed, but TUG performance improved only when NMES was combined with voluntary exercise (Benavent-Caballer et al., 2014).

Although the pathologies of muscle ageing and disuse differ, they are often strongly associated. Immobilisation often occurs in the elderly as the decline in their functional capacity increases propensity for falls and fracture (Dirks et al., 2014; Marks, 2011). NMES applied to support the functional recovery of elderly women after hip fracture surgery resulted in a faster recovery of indoor mobility (Lamb et al., 2002). Moreover, the follow-up assessment after an additional six weeks found that NMES induced a longer-term effect on functional rehabilitation, showing an improvement in walking speed, as well as postural stability and muscle power.

Numerous studies have demonstrated that NMES induces a significant attenuation of muscle atrophy during or after immobilisation, however, owing to the high variability of procedures and protocols, direct comparisons cannot be reliably performed. Collectively, the available data demonstrates that NMES exerts positive effects on functional rehabilitation.

## 6 Conclusions

This review details the adaptations to NMES at the individual muscle fibre level and within the peripheral motor nervous system. Although this review is not exhaustive with regards to the multiple pathologies to which NMES has been applied, the majority of findings indicate that NMES exerts positive effects on both neural and muscular remodelling in terms of morphological, molecular and functional aspects in the elderly. The mechanistic knowledge obtained thus far is largely derived from animal studies, with little human data available, indicating the potential direction of future research.

## CRediT authorship contribution statement

**Yuxiao Guo:** Conceptualization, Methodology, Writing - original draft, Writing - review & editing, Visualization. **Bethan E Phillips:** Conceptualization, Methodology, Writing - review & editing, Visualization. **Philip J Atherton:** Conceptualization, Methodology, Writing - review & editing, Visualization, Supervision. **Mathew Piasecki:** Conceptualization, Methodology, Writing - review & editing, Visualization, Supervision.

## Declaration of Competing Interest

The authors declare that they have no known competing financial interests or personal relationships that could have appeared to influence the work reported in this paper.
